# Cannabis recreativo: Perfil de los cannabinoides presentes en muestras de marihuana suministradas por población consumidora

**DOI:** 10.18294/sc.2023.4385

**Published:** 2023-03-30

**Authors:** Santiago Gómez Velásquez, Ángela María Amaya Heredia, Santiago Bedoya Moncada, Juan Esteban Patiño González, Jorge Ariel Martínez Ramírez

**Affiliations:** 1 Magíster en Salud Pública. Secretaría de la Juventud de Medellín. Estudiante del Doctorado en Epidemiología y Bioestadística, Universidad CES, Medellín, Colombia. sagomez@ces.edu.co Universidad CES Universidad CES Medellín Colombia sagomez@ces.edu.co; 2 Química farmacéutica. Universidad Nacional de Colombia, Bogotá, Colombia. aamayah@unal.edu.co Universidad Nacional de Colombia Universidad Nacional de Colombia Bogotá Colombia aamayah@unal.edu.co; 3 Politólogo. Secretario de la Juventud de Medellín, Medellín, Colombia. santiago.bedoyamon@gmail.com Secretario de la Juventud de Medellín Secretario de la Juventud de Medellín Medellín Colombia santiago.bedoyamon@gmail.com; 4 Magíster en Psicología. Secretaría de la Juventud de Medellín, Medellín, Colombia. juan.patinog@udea.edu.co Secretario de la Juventud de Medellín Secretario de la Juventud de Medellín Medellín Colombia juan.patinog@udea.edu.co; 5 PhD en Toxicología Forense. Profesor, Departamento de Farmacia, Facultad de Ciencias, Universidad Nacional de Colombia, Bogotá, Colombia. jamartinezra@unal.edu.co Universidad Nacional de Colombia Facultad de Ciencias Universidad Nacional de Colombia Bogotá Colombia jamartinezra@unal.edu.co

**Keywords:** Cannabis, Cannabinoides, Tetrahydrocannabinol, Cannabidiol, Colombia

## Abstract

El cannabis o marihuana es una de las sustancias psicoactivas más consumida en todo el mundo, por lo que conocer la composición y el tipo de cannabis que se comercializa en los entornos urbanos es un insumo necesario para el diseño de políticas en salud pública sustentadas en la evidencia científica. Este estudio caracterizó los principales fitocannabinoides de muestras de marihuana (cigarrillos o cogollos) obtenidas en áreas urbanas y rurales de la ciudad Medellín, en octubre de 2021. Se realizó un muestreo no probabilístico a conveniencia en el que se recolectaron 87 muestras de marihuana donadas por consumidores en diferentes puntos de recolección en toda la ciudad, aplicando las técnicas de cromatografía de gases masas e ionización de llama para la caracterización de los fitocanabinoides. Se encontró el tetrahidrocannabinol como el constituyente principal de la marihuana circulante en Medellín, donde el 67,8% de las muestras presentaba un rango toxicológico alto o superior para THC; lo anterior en un contexto donde el mercado desregulado limita la posibilidad que tienen los consumidores en la práctica de calibrar o decidir la concentración de cannabinoides en sus dosis.

## INTRODUCCIÓN

La *Cannabis Sativa* L también conocida como cannabis o marihuana, una planta milenaria que hoy en día se cultiva alrededor de todo el mundo, ha sido ampliamente empleada a nivel medicinal y recreacional. Según el “Código de los Estados Unidos”, el término cannabis o marihuana corresponde a todas las partes de la planta *Cannabis L.*, ya sea que crezcan o no, las semillas, la resina extraída de cualquier parte de la planta, y todo compuesto, manufactura, sal, derivado, mezcla o preparación de dicha planta, sus semillas o resina^1^. Dicha planta pertenece a la familia Cannabaceae y hoy en día, de acuerdo a las más recientes clasificaciones, se sabe que tiene alrededor de 170 especies^2^. La especie Cannabis es la más conocida, la cual tiene la capacidad de sintetizar alrededor de 565 sustancias, 120 de las cuales, corresponden a moléculas de 21 átomos de carbonos con un esqueleto terpenofenólico llamadas fitocannabinoides, siendo el delta 9 tetrahidrocannabinol (Δ9 -THC o THC) el cannabinoide que se encuentra en mayor proporción en la planta y el responsable de la gran mayoría de los efectos psicoactivos del cannabis. Otros fitocannabinoides, que también presentan actividad psicotrópica y que están en menor proporción en el material vegetal, son la cannabivarina (THCV) y el CBN, este último evaluado en el presente estudio como marcador de oxidación del THC. Otros cannabinoides que no presentan actividad psicoactiva y que se pueden encontrar en la planta en diferentes concentraciones dependiendo de la cepa son cannabidiol (CBD), cannabicromeno (CBC), y canabigerol (CBG)^3^^,^^4^. 

Es originaria de los ambientes tropicales de Asia centro-oriental y ha sido utilizada por los humanos desde la antigüedad, adaptándose a diferentes condiciones climáticas, hoy en día es un cultivo que se extiende en buena parte del mundo^5^. Cuando el cannabis es consumido, activa el sistema de receptores y neurotransmisores dentro del organismo llamado sistema endocannabinoide (SEC). En el caso del THC, este se une a receptores CB1 y CB2 generando un amplio rango de efectos, algunos de ellos más deseados que otros. Por ejemplo, puede ayudar a reducir el dolor y mejorar el apetito; sin embargo, al mismo tiempo puede causar paranoia y ansiedad. Por su parte el CBD disminuye la señalización de los endocanabinoides y esta respuesta a su vez depende de la dosis al unirse al grupo alostérico del receptor CB1 alterando la potencia de otros ligandos primarios, produciendo efectos antagónicos^6^^,^^7^.

Los efectos psicotrópicos más destacables del uso recreacional del THC informados por usuarios son: placer, relajación, felicidad y aumento de la percepción sensorial entre otros^8^. Sin embargo, estos efectos son acompañados de efectos indeseables sobre el sistema nervioso central, el sistema respiratorio, el sistema cardiovascular y algunas condiciones psiquiátricas caracterizados por depresión, sedación, síntomas psicóticos, incremento de la actividad cardiovascular, deterioro de enfermedades mentales preexistentes y síntomas de dependencia^9^.

La cantidad de THC en muestras de cannabis puede disminuir por interconversión a cannabinol (CBN), un cannabinoide que no está presente en la planta, con muy bajo poder psicoactivo y que se generada por calentamiento u oxidación del material vegetal. Altas concentraciones de CBN en las muestras de cannabis pueden indicar un almacenamiento prolongado o mala manipulación de la muestra^10^. 

En cuanto al cannabinoide CBD, extractos vegetales y derivados ricos en este cannabinoide han sido empleados en terapias para dolor crónico, enfermedades respiratorias, algunos tipos de cáncer, y riesgo cardiometabólico entre otras patologías^11^. La variedad en los efectos depende de la cantidad y concentración de cannabinoides presente en material vegetal y derivados utilizado por los usuarios. 

Tradicionalmente y dependiendo de la cantidad de estos componentes, el *Cannabis sativa* puede clasificarse en tres quimiotipos. El quimiotipo I es rico en el cannabinoide psicotrópico THC; la gran mayoría de los cultivares modernos pertenecen a esta categoría; estas plantas son muy apreciadas por los consumidores con fines recreativos que buscan “colocarse”, y por quienes consumen marihuana con fines holísticos. El quimiotipo II ofrece una proporción de THC y CBD equilibrada; los cultivares con este equilibrio están teniendo mucho éxito tanto entre consumidores con fines recreativos como holísticos; produce un efecto psicotrópico fuerte, pero una cantidad similar de CBD atenúa la influencia del THC y podría reducir sus efectos psicotrópicos perjudiciales. El quimiotipo III es rico en CBD y tiene un contenido bajo de THC, por eso, estas variedades apenas producen efectos psicotrópicos; para algunos consumidores recreativos y holísticos, este efecto lúcido resulta muy útil y funcional^4^. Aunque esta clasificación no es válida desde el punto de vista taxonómico de la semilla, es práctica desde el punto de vista analítico, farmacológico y legal (en el caso de Colombia) ya que rápidamente se puede ubicar el tipo de muestra de acuerdo a su concentración y asociarla a un riesgo toxicológico y legal. En Colombia se considera material psicoactivo cuando la concentración de THC supera el 1%^12^.

Los efectos psicotrópicos de los cannabinoides presentes en la marihuana, la convierten en una de las sustancias psicoactivas más consumida en todo el mundo, tanto en contextos de legalización como de prohibición del consumo. La evidencia indica que las prevalencias de consumo se han mantenido en aumento durante las últimas décadas, con tasas que registran consumos superiores en jóvenes y adultos jóvenes, donde el uso de la sustancia permanece vigente en diferentes territorios independiente del marco normativo que la regule^13^^,^^14^.

Algo similar ocurre en el continente americano, la información más reciente revela que el consumo de marihuana ha aumentado en ocho países de los once que reportaron datos; de acuerdo con los informes de la Organización de Estados Americanos (OEA) el consumo se está generando en edades cada vez más tempranas y la percepción general de riesgo asociada al consumo ha disminuido, con cambios en los hábitos y formas de consumo^15^. 

En Colombia, la última Encuesta Nacional de Consumo de Sustancias Psicoactivas realizada en 2019 por el Departamento Administrativo Nacional de Estadística (DANE), obtuvo como hallazgo -compatible con las tendencias a nivel global y continental- que la mayor prevalencia vida de consumo en sustancias de uso ilícito se presentó para la marihuana con un 8%. Es necesario destacar que los jóvenes colombianos, coincidente con las tendencias etarias en el continente, también registran un inicio cada vez más temprano del consumo de marihuana, y una visible inclinación a experimentar con una evidente percepción de riesgo leve^16^. De igual manera, la evidencia indica sin ambigüedades un incremento en el consumo de las sustancias catalogadas como ilegales (marihuana) y una disminución en el consumo de las sustancias legales (tabaco) en población colombiana^15^^,^^17^, el enfoque de reducción de oferta que se ha implementado por años desde el Estado colombiano no ha reducido el consumo; las investigaciones sobre este particular establecen, de forma consistente a lo largo de tres décadas de estudio, que las personas colombianas perciben una ausencia casi total de obstáculos para acceder a sustancias como la marihuana^18^. 

Por otra parte, la literatura científica pone de manifiesto que, desde 1992 y hasta la última encuesta nacional de 2019, la ciudad de Medellín es una de las regiones en las que se presentan prevalencias de uso de sustancias psicoactivas superiores a las del promedio nacional^18^. Este resultado es corroborado por estudios locales como “El Análisis de la Situación de Salud 2005-2015” publicado por la Alcaldía de la ciudad, en el que afirma: “El municipio de Medellín presenta mayor prevalencia de vida que el país en el consumo de alcohol, tabaco, marihuana, cocaína, bazuco, tranquilizantes, estimulantes, heroína, éxtasis y en general de cualquier sustancia legal, ilegal o de uso indebido”. Asimismo, los jóvenes de la ciudad presentan prevalencias de vida de consumo de marihuana superiores, en comparación con grupos poblacionales en otros rangos de edad^19^^,^^20^. 

Como puede colegirse con lo expuesto hasta aquí, las acciones de las instituciones nacionales y municipales para afectar la demanda y uso de sustancias psicoactivas, en general, y de la marihuana, en particular, no han generado cambios significativos en los patrones de consumo. Adicionalmente, los diferentes estudios alrededor del abuso de cannabis se centran básicamente en la frecuencia de consumo^21^, dejando de lado la composición y la concentración de cannabinoides que pueden tener las diferentes muestras. Esta situación es preocupante si se tiene en cuenta que el riesgo toxicológico definitivamente cambia de acuerdo con la concentración del principal componente psicoactivo de la planta. No obstante, los debates en torno a la legalización del consumo recreacional de la marihuana y su uso medicinal siguen marcando las agendas políticas tanto en Colombia como en diferentes países de la región, lo que indicaría una transición del paradigma prohibicionista hacia estrategias médico-preventivas, de salud pública y de reducción de daños y riesgos^22^.

Identificar los tipos de cannabinoides presentes en muestras de marihuana que circulan en los entornos urbanos es una acción indispensable para el desarrollo de estrategias de atención integral en salud pública y medicina especializada, que puedan orientar intervenciones preventivas frente a las problemáticas asociadas con el consumo de sustancias psicoactivas; entregando de manera simultánea la posibilidad de fortalecer o resignificar el enfoque de los programas de promoción y prevención para el abordaje de las conductas de riesgo, sustentando su diseño y ejecución en la evidencia científica.

En Colombia, a través de la Ley 1787 del 2016, se creó un marco regulatorio para permitir el acceso seguro e informado al uso médico y científico del cannabis y sus derivados. En este sentido, a través del Decreto 613 del 2017, emitido por el Ministerio de Salud y Protección Social, se considera que un material de cannabis es psicoactivo si la concentración de THC es mayor a 1% en peso seco, indicando que para los derivados de cannabis se deberá como mínimo cuantificar los cannabinoides THC como componente psicoactivo, CBD como componente medicinal y CBN como indicador de degradación del THC. De acuerdo a esta reglamentación, este trabajo tuvo como objetivo principal conocer cuál es la concentración de THC, CBD y CBN presente en muestras de cannabis (material vegetal) recolectadas de manera voluntaria en seis comunas y dos corregimientos del distrito de Medellín, obteniendo una caracterización sociodemográfica general del consumo con fines recreacionales.

## MATERIALES Y MÉTODOS

Estudio de tipo transversal con alcance analítico y captación prospectiva del dato, en el cual se realizó un muestreo no probabilístico para la captación de usuarios de cannabis en la ciudad de Medellín, que de forma voluntaria estuvieran dispuestos a donar la sustancia consumida como muestra para el análisis. Esta investigación no presentó ningún riesgo para los participantes según lo establece la Resolución 8430 de 1993, habiendo acatado lo concerniente al código bioético en el proceso de recolección de la información; sin embargo, a cada participante se le entregó un aplicativo tipo encuesta con la finalidad de recolectar información sociodemográfica y de frecuencias de consumo, sin identificar, ni indagar sobre aspectos sensitivos de su conducta, el cual fue contestado sin su firma, pero con consentimiento de tipo verbal, teniendo en cuenta que los consumidores de este tipo de sustancias son temerosos a ser identificados, estigmatizados y posiblemente judicializados. La recolección y posterior trabajo con las muestras fue avalado por el Centro Internacional de Estudios Estratégicos contra el Narcotráfico (CIENA) de la Policía Nacional y por el Fondo Nacional de Estupefacientes. Con el ánimo de preservar en su totalidad el anonimato de los participantes del estudio, ninguna de estas dos entidades tuvo contacto directo ni indirecto con alguno de ellos, evitando prácticas que pudieran percibirse como de identificación o rastreo.

### Recolección de las muestras

La recolección de las muestras estuvo a cargo de la empresa Consultoría Especializada en Drogas, Salud & Sociedad (CEDSS) contratada por la Universidad Nacional de Colombia, Sede Bogotá. Todas las muestras fueron recogidas en el distrito de Medellín durante el mes de octubre del año 2021, mediante 22 intervenciones en diferentes eventos sociales dirigidos a población joven, en los cuales fue instalado un puesto fijo de análisis y atención, con la finalidad de que los usuarios de cannabis se acercaran de forma voluntaria a realizar la donación de la sustancia. En cada momento de recolección se siguió un protocolo detallado para garantizar la estandarización y calidad en el proceso de recolección y a cada usuario se le pidió llenar de manera potestativa un aplicativo que permitió recoger información de la muestra y datos sociodemográficos generales. A cada usuario se le entregó la información respectiva sobre la investigación, sus objetivos y el uso estrictamente académico e investigativo de la información resultante, solicitándole finalmente su consentimiento verbal para diligenciar el aplicativo y donar voluntariamente la muestra. Durante las diferentes jornadas de recolección se llevaron a cabo cuatro tipos de muestreos: muestreo en cadena o bola de nieve, muestreo por conveniencia, muestreo por cuotas y muestreo por participantes voluntarios o autoseleccionado. Una vez que se recolectaron todas las muestras, estas fueron trasladadas por funcionarios del “Centro internacional de estudios estratégicos contra el narcotráfico” (CIENA) al laboratorio de Análisis Instrumental Farmacéutico del Departamento de Farmacia de la Universidad Nacional de Colombia, donde se llevaron a cabo los respectivos análisis cromatográficos.

### Tipo de muestras y preparación para su análisis

Como criterio de selección en la recolección de las muestras se definió que fueran exclusivamente material vegetal. El resto fueron excluidas de este estudio. Las muestras se recolectaron en las comunas de Laureles-Estadio, La Candelaria, Poblado, San Javier, Guayabal, Robledo y los corregimientos de San Antonio de Prado y Santa Elena del distrito de Medellín durante el mes de octubre de 2021.

Cada una de las muestras de material vegetal de cannabis fueron maceradas hasta obtener un tamaño de partícula homogéneo. Una porción (50 mg) del macerado fue empleado para llevar a cabo la extracción de los cannabinoides suspendiéndolo en 1,5 mL de etanol en un vial eppendorf seguido de vortex durante tres minutos, sonicación durante cinco minutos y centrifugación a 9.000 rpm durante cinco minutos. El sobrenadante fue filtrado en vial para cromatografía, empleando una membrana de 0,45 µm y 1 µL fue inyectado en cada uno de los cromatógrafos de gases.

### Técnica de análisis químico

Para la identificación de los diferentes cannabinoides, en todas las muestras se empleó cromatografía de gases con detector de masas e impacto electrónico (GC-MS/EI) y para la cuantificación, cromatografía de gases con detector de ionización de llama (FID). Ambas metodologías analíticas fueron validadas de acuerdo con las guías de validación de la Oficina de las Naciones Unidas contra la Droga y el Delito (UNODC)^23^.

Los parámetros para la identificación de los diferentes cannabinoides, mediante la metodología analítica GC-MS/EI, fueron tomados del documento de la UNODC “Métodos recomendados para la identificación y el análisis del cannabis y los productos del cannabis”^24^.

GC-MS/EI. El Sistema empleado para la identificación de los diferentes fitocannabinoides empleó un cromatógrafo de gases Thermo TRACE 1300 con Detector ISQ QD y una columna analítica capilar TR-5MS 30 m x 0,25 mm i.d x 0,25 µm. Las temperaturas del inyector, detector y línea de transferencia fueron 290°C, 230°C y 280°C respectivamente. La inyección fue hecha en modo split 20:1 y el horno fue programado iniciando en 240°C durante un minuto, con un incremento de 12°C/min hasta 270°C por cinco minutos. Para la evaluación de los datos se empleó el software Chromeleon® con la librería de masas NIST 2007.

GC-FID. La cuantificación de los fitocannabinoides en el material vegetal fue llevada a cabo usando un cromatógrafo de gases Shimadzu GC 2010 plus equipado con un automuestreador Shimadzu AOC-20i y una columna analítica capilar (30 m x 0,25 mm x 0,25 µm, Restek, Bellefonte, Pennsylvania, US). La temperatura del detector y el inyector fueron mantenidas a 290 °C y 300 °C, respectivamente. Un 1 µL del extracto de cannabis disuelto en etanol al 99% fue inyectado en modo split 10:1. El horno fue programado iniciando en 200 °C durante dos min con un incremento de 10 °C/min hasta 260°C por siete minutos, con un tiempo total de corrida de 15 minutos. La cuantificación fue llevada a cabo para THC, CBD y CBN con tetracosano como estándar interno en una concentración de 100 ppm. Para la evaluación de los datos se empleó el software Lab Solutions vr 5,52 de Shimadzu.

### Sustancias químicas y reactivos

Material de referencia “THC cannabinoids mixture-3” con delta-9-tetrahidrocannabinol (Δ9-THC), cannabinol (CBN) y cannabidiol (CBD) 1 mg/mL - Cerilliant Corporation (Round Rock, TX) y Tetracosano del 99% adquiridos de Sigma/Merck, Colombia, etanol grado analítico al 99% PanReac. Solución de trabajo de cannabinoides en concentración de 100 µg/mL.

### Análisis estadístico

El análisis de la información se realizó utilizando el software estadístico de código abierto Jamovi 2.3.21; las variables de tipo categórico fueron analizadas calculando frecuencias absolutas y relativas. Para las variables cuantitativas se usaron estadísticas descriptivas de tendencia central (media y mediana) y de dispersión (desviación estándar y rango intercuartil).

## RESULTADOS

En total se recogieron 87 muestras de cannabis correspondientes a material vegetal con igual número de aplicativos. La cantidad donada en promedio para este material fue de 282,8 mg, con un rango que va desde 29,1 a 1.037,4 mg*.*

Con respecto a las características sociodemográficas de las personas donantes de las muestras, en la Tabla 1 se puede observar que 22 fueron mujeres, 63 hombres, mientras 2 personas no respondieron la categoría sexo; que la mayoría de las personas que contribuyeron con la donación de las muestras tenía formación profesional o de postgrado y se ubicaba en un rango de edad entre los 29 y 59 años. Destaca además que el 70% de las personas donantes pertenecían al nivel socioeconómico medio (estrato 3-4) o alto (estrato 5-6) de acuerdo con la estratificación socioeconómica en Colombia^25^. El 80,5% de los consumidores llevaban más de 5 años consumiendo la sustancia.

**Tabla 1:** Caracterización de las personas usuarias que donaron las muestras (n=87). Comunas y corregimientos de Medellín, Colombia, 2021.

Características	Total		Mujeres		Hombres	
	n	%	n	%	n	%
Formación						
Ninguna	3	3,5	0	0,0	3	4,8
Primaria	1	1,2	0	0,0	1	1,6
Secundaria	15	17,2	1	4,6	14	22,2
Técnico/Tecnólogo	17	19,5	4	18,2	13	20,6
Profesional	40	46,0	17	77,3	23	36,5
Posgrado	8	9,2	0	0,0	8	12,7
No responde	3	3,5	0	0,0	1	1,6
Edad						
18-28	29	33,3	7	31,8	22	34,9
29-59	55	63,2	15	68,2	40	63,5
> 60	1	1,2	0	0,0	1	1,6
No responde	2	2,3	0	0,0	0	0,0
Nivel socioeconómico*						
Bajo	24	27,6	2	9,1	22	34,9
Medio	53	60,9	19	86,3	34	54,0
Alto	8	9,2	1	4,5	7	11,1
No responde	2	2,3	0	0,0	0	0,0
Tiempo de consumo						
1 a 2 años	2	2,3	0	0,0	2	3,2
3 a 5 años	11	12,6	2	9,1	9	14,3
Más de 5 años	70	80,5	19	86,4	49	77,8
No responde	4	4,6	1	4,6	3	4,8

Fuente: Elaboración propia. Nota: Del total de personas usuarias que donaron las muestras, dos personas no respondieron la categoría sexo. *Corresponde a la estratificación socioeconómica de los inmuebles residenciales de un municipio, que se hace en atención al Régimen de los Servicios Públicos Domiciliarios en Colombia según la Ley 142 de 1994.

### Análisis cromatográfico

Las 87 muestras recolectadas en el marco del Programa de Salud Pública Juvenil de la Secretaría de la Juventud de Medellín se obtuvieron en seis de las 16 comunas del área urbana de la ciudad, y en dos de los cinco corregimientos que conforman la zona rural. De acuerdo con la Tabla 2, los territorios urbanos aportaron el 83,9% (73) de las muestras y la zona rural el 16,1% (14).

**Tabla 2:** Lugar de recolección de las muestras de cannabis de material vegetal (n=87). Comunas y corregimientos de Medellín, Colombia, 2021.

Comuna y corregimientos	n	%
Laureles-estadio	27	31,0
La candelaria	18	20,7
Poblado	12	13,8
Santa Elena	8	9,8
San Javier	7	8,1
San Antonio de prado	6	6,9
Guayabal	5	5,8
Robledo	4	4,6

Fuente: Elaboración propia.

De las seis comunas en las que se captaron muestras, las comunas ubicadas sobre el costado occidental (Laureles, Guayabal, Robledo y San Javier) aportaron el 58,9%. Por su parte, las comunas ubicadas al oriente de la ciudad (La Candelaria y Poblado) contribuyeron con el 41,1%. El corregimiento de San Antonio de Prado suministró el 42,8% de las muestras procedentes de la zona rural. El número de muestras reunidas y su dispersión espacial permitieron obtener un panorama amplio de los cannabinoides presentes en la marihuana que se consume en la ciudad de Medellín.

### Análisis de las muestras

#### GC-MS/EI

El análisis cualitativo por GC-MS/EI permitió la detección de siete fitocanabinoides ordenados de acuerdo con su incidencia en las diferentes muestras (Tabla 3). En su orden se encuentran tetrahidrocannabinol (THC), el Cannabigerol (CBG), el Cannabinol (CBN), el Cannabicromeno (CBC), y el Delta-9-Tetrahidrocannabivarina; Cannabidiol (CBD) y Cannabidivarol (CBDV).

**Tabla 3:** Cannabinoides presentes en las muestras de cannabis de material vegetal (n=87). Comunas y corregimientos de Medellín, Colombia, 2021.

Cannabinoides	n	%
THC (tetrahidrocannabinol)	87	100,0
Cannabigerol (CBG)	78	89,5
Cannabinol (CBN)	67	77,0
Cannabicromeno (CBC)	63	72,4
Delta-9-Tetrahidrocannabivarina	47	54,0
Cannabidiol (CBD)	13	14,9
Cannabidivarol (CBDV)	2	2,2

Fuente: Elaboración propia.

La estructura y composición de cada uno de los fitocannabinoides se puede observar en la Tabla 4. La presencia de THC en el 100% de las muestras y de CBG en 78 de ellas, sigue un patrón lógico, teniendo en cuenta que el mayor componente de los cannabinoides en material vegetal es de THC y por su parte el CBG proviene del CBG ácido, primer fitocannabinoide que se biosintetiza después de la formación de ácido olivetólico, siendo el precursor de los demás cannabinoides en la planta^26^. No obstante, y a pesar de que el THC presentó una media de 89,4% de la composición total de cannabinoides de las 87 muestras, el CBD cannabinoide terapéutico no psicoactivo tuvo una media de 37,1%, porcentaje promedio nada despreciable en la composición total de las 13 muestras en que estuvo presente. La presencia promedio de los demás cannabinoides (CBG, CBN, CBC, CBDV y el Delta-9-Tetrahidrocannabivarina) fue inferior al 2,3%.

En cuanto a su actividad psicotrópica^27^, en este estudio se encontró THC y CBN (baja actividad), como cannabinoides psicoactivos y CBG, CBC, CBD y el CBDV como componentes no psicoactivos. En comparación con otros cannabinoides, el THC en su composición porcentual fue muy superior. Por ello, al estudiar la composición del cannabis de la ciudad de Medellín, el THC se debe considerar como el contribuyente principal del tipo de efecto que los consumidores esperan de la sustancia. El CBN, otro componente menos psicoactivo del cannabis y diez veces menos potente que el THC^28^, estuvo presente en el 77,0% de las muestras (Tabla 3). Sin embargo, su concentración en las muestras no supera el 1% y por consiguiente se puede afirmar que todas las 87 muestras analizadas correspondieron a material vegetal fresco y con baja manipulación.

En cuanto a los cannabinoides no psicoactivos, el CBD fue identificado en 13 muestras con un promedio de presencia porcentual significativa comparada con los demás cannabinoides (37,1%). El CBD se diferencia estructuralmente del THC por la presencia de un doble enlace de carbono y un grupo hidroxilo que previene los efectos deletéreos de las altas dosis de THC, moderando sus efectos psicoactivos, con propiedades medicinales^29^. Asimismo el CBG fue identificado en 78 muestras con una presencia porcentual promedio del 2,3% (Tabla 4). Estos dos cannabinoides, al actuar por un mecanismo diferente al del THC, cuentan potencialmente con ciertos efectos antiinflamatorios, analgésicos, antipsicóticos, antiisquémicos, antiepilépticos y ansiolíticos que hoy en día están siendo ampliamente estudiados^30^^,^^31^.

**Tabla 4:** Presencia porcentual de cada cannabinoide encontrado con respecto al total de cannabinoides cuantificados en las muestras de cannabis de material vegetal (n=87). Comunas y corregimientos de Medellín, Colombia, 2021

Cannabinoide	Estructura molecular	Muestras positivas	Composición		
			Media ± DE	Mediana	(Q1 - Q3)
THC (tetrahidrocannabinol)	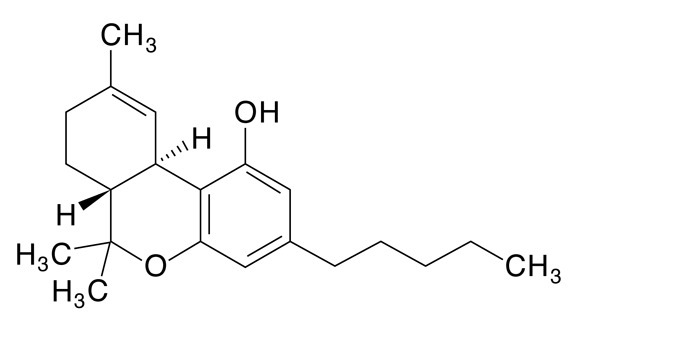	87	89,4 ± 15,8	94,9	92,1 - 95,9
CBG Cannabigerol	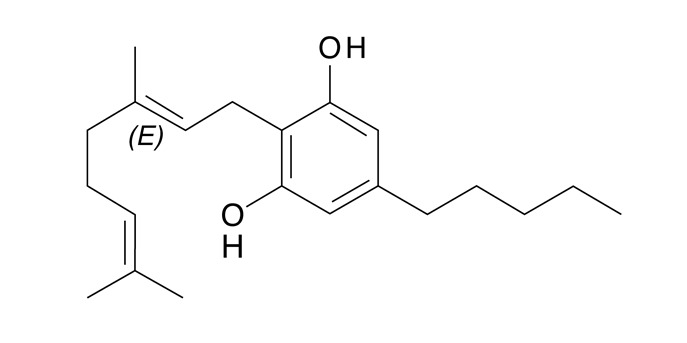	78	2,3 ± 1,7	1,9	1,5 - 2,5
CBN Cannabinol	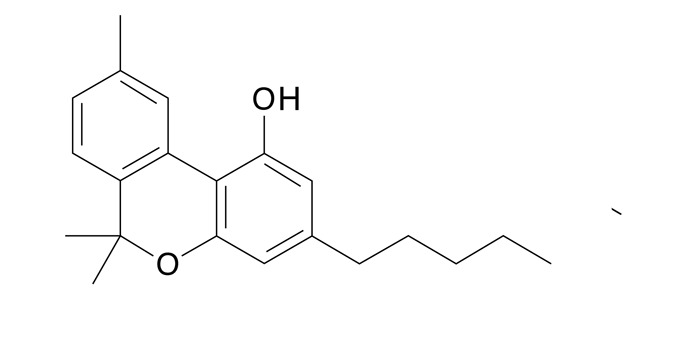	67	1,8 ± 2,0	1,1	0,8 - 1,9
CBC Cannabicromeno	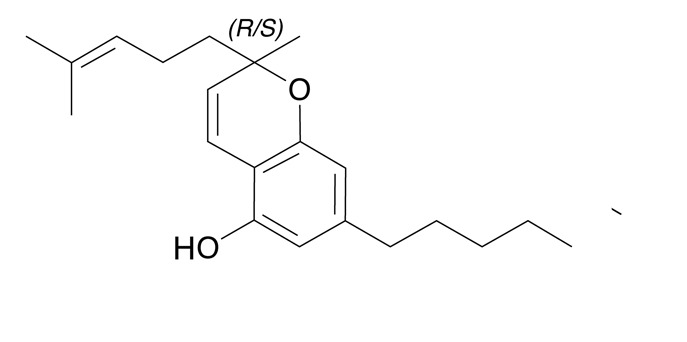	63	1,8 ± 1,1	1,4	1,2 - 2,0
Delta-9-Tetrahidrocannabivarina	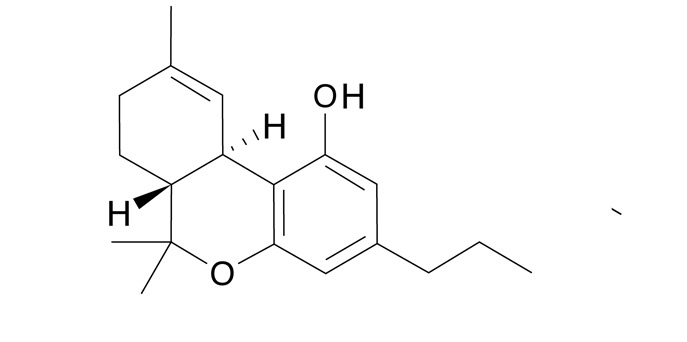	47	0,5 ± 0,4	0,4	0,3 - 0,6
CBD Cannabidiol	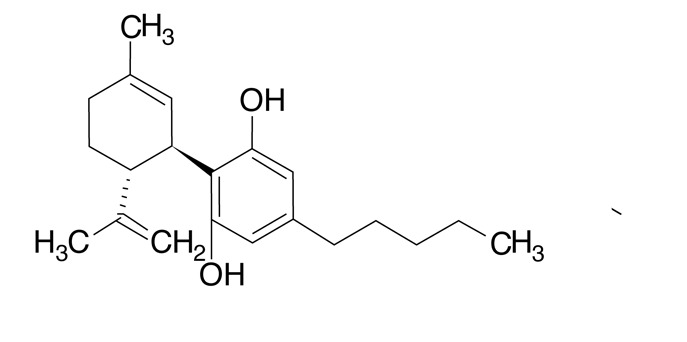	13	37,1 ± 26,1	51,3	6,9 - 57,0
CBDV Cannabidivarol	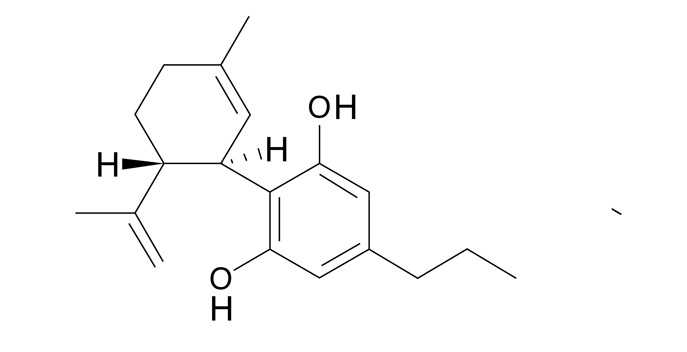	2	0,9 ± 0,9	0,9	0,6 - 1,3

Fuente: Elaboración propia.

En la Tabla 5 se reporta el quimiotipo y el promedio de concentración del THC, mientras en la Tabla 6 se muestra el porcentaje de cada uno de los tres cannabinoides más relevantes en este estudio.

**Tabla 5:** Quimiotipo y riesgo toxicológico en función del THC presente en las muestras de cannabis analizadas (n=87). Comunas y corregimientos de Medellín, Colombia, 2021.

Comunas y corregimientos	Quimiotipo				Riesgo toxicológico (THC)*									
	I		II		Extremadamente alto		Muy alto		Alto		Medio		Bajo	
	n	%	n	%	n	%	n	%	n	%	n	%	n	%
San Javier	7	100,0	0	0,0	0	0,0	0	0,0	2	28,6	3	42,9	2	28,6
Robledo	4	100,0	0	0,0	1	25,0	0	0,0	2	50,0	1	25,0	0	0,0
La Candelaria	17	94,4	1	5,6	0	0,0	3	16,7	9	50,0	5	27,8	1	5,6
Laureles-Estadio	25	92,6	2	7,4	0	0,0	8	29,6	13	48,2	4	14,8	2	7,4
Poblado	12	100,0	0	0,0	1	8,3	3	25,0	5	41,7	1	8,3	2	16,7
Guayabal	5	100,0	0	0,0	0	0,0	3	60,0	1	20,0	1	20,0	0	0,0
San Antonio de Prado	6	100,0	0	0,0	0	0,0	1	16,7	5	83,3	0	0,0	0	0,0
Santa Elena	3	37,5	5	62,5	0	0,0	0	0,0	2	25,0	2	25,0	4	50,0
Total	79	90,8	8	9,2	2	2,3	18	20,7	39	44,8	17	19,5	11	12,6

Fuente: Elaboración propia.

*Rango de concentración de THC - Riesgo Toxicológico^32^: bajo: contenido de THC entre 1,0% - 5,0%; medio: contenido de THC entre 5,0% - 10,0%; alto: contenido de THC entre 10,0% - 15,0%; muy alto: contenido de THC entre 15,0% - 20,0%; extremadamente alto: contenido de THC entre 20,0% - 25,0%.

**Tabla 6:** Cuantificación del THC, CBD y CBN presentes en las muestras de cannabis analizadas (n=87). Comunas y corregimientos de Medellín, Colombia, 2021.

Comunas y corregimientos	Peso muestra (mg)	THC (% p/p)	CBD (% p/p)	CBN (% p/p)	CBD+THC+CBN (% p/p)
	Media ± DE	Media ± DE	Media ± DE	Media ± DE	Media ± DE
San Javier	101,2 ± 37,6	7,6 ± 3,1	0,2 ± 0,1	0,3 ± 0,2	8,1 ± 3,2
Robledo	231,3 ± 115,7	14,7 ± 6,6	0,4 ± 0,3	0,1 ± 0,1	15,2 ± 6,6
La Candelaria	380,6 ± 320,4	11,0 ± 3,7	0,6 ± 1,4	0,1 ± 0,2	11,7 ± 3,2
Laureles-Estadio	278,7 ± 177,1	12,4 ± 4,2	0,8 ± 2,0	0,1 ± 0,1	13,2 ± 3,8
Poblado	260,3 ± 147,2	12,9 ± 5,3	0,2 ± 0,1	0,3 ± 0,6	13,4 ± 5,6
Guayabal	158,7 ± 78,6	13,1 ± 4,2	0,2 ± 0,0	0,3 ± 0,1	13,5 ± 4,3
San Antonio de Prado	198,9 ± 131,0	13,8 ± 2,8	0,2 ± 0,0	0,2 ± 0,1	14,1 ± 2,9
Santa Elena	435,6 ± 295,0	6,1 ± 4,1	4,4 ± 3,5	0,0 ± 0,0	10,5 ± 2,3
Total	282,8 ± 223,4	11,5 ± 4,7	0,8 ± 2,0	0,1 ± 0,3	12,4 ± 4,2

Fuente: Elaboración propia.

De acuerdo con la legislación actual colombiana, todas las muestras cuantificadas estarían clasificadas como material psicoactivo debido a que la concentración de THC supera el 1% p/p (Tabla 5). La gran mayoría de las muestras cuantificadas (90,8%) corresponden a un quimiotipo I rico en el cannabinoide THC, por lo que estas principalmente tienen efectos psicotrópicos. Un porcentaje del 9,2%, correspondiente a 8 muestras, presentó quimiotipo II rico en CBD (mayor contenido de CBD que de THC), por lo cual es posible presumir que estas poseen menos efectos psicotrópicos sobre el organismo al contrarrestarse estos últimos con los efectos terapéuticos generados por las altas concentraciones del cannabinoide medicinal. Específicamente, las 8 muestras ricas en CBD reportaron contenidos de este cannabinoide en el rango de 4,7 a 9,6% p/p. Las comunas en las cuales se recolectó mayor cantidad de muestras de material vegetal de cannabis fueron Laureles-Estadio, La Candelaria y Poblado. De manera general, la mayor parte de las muestras (44,8%) presenta riesgos toxicológicos altos con relación a la concentración de THC presente.

Como muestra la Tabla 6, en promedio, se encontraron concentraciones (% p/p) de THC en un rango de 6,1-14,7% con una media del 11,5%, las concentraciones de CBD de 0,2-4,4% con una media del 0,8%, y las concentraciones de CBN de 0,0-0,3% con una media del 0,1%, La concentración de la suma total de los tres cannabinoides mencionados tuvo una media del 12,4%.

## DISCUSIÓN

A pesar de que la evidencia científica ha demostrado que el consumo agudo de cannabis aumenta la inflamación de las vías respiratorias, destruye el tejido pulmonar, y que simultáneamente hay estudios que muestran cómo el uso crónico de cannabis resulta en un mayor riesgo de enfermedades crónicas como bronquitis, de enfisema, inflamación respiratoria crónica y deterioro de la función respiratoria^33^, fumar marihuana sigue siendo un práctica que va en aumento, tanto en Medellín como en diferentes ciudades de la región^18^^,^^34^^,^^35^^,^^36^^,^^37^. Todos los efectos adversos que se presentan en vías respiratorias y tejido pulmonar están estrechamente relacionados con la principal manera de consumo de la marihuana. Cuando esta es consumida por vía pulmonar, las concentraciones sanguíneas de THC se alcanzan entre siete y diez minutos después del consumo, con generación de efectos máximos, los cuales están sujetos a variabilidad interindividual, en 20 a 30 minutos, y se extienden hasta por un máximo de tres a cuatro horas^38^. 

Al caracterizar los componentes de las muestras de marihuana obtenidas, se puede destacar varios hallazgos relevantes. En primer lugar, el contenido de cannabinoides no es constante en todas las muestras, lo cual indica que la oferta de cannabis en el Distrito de Medellín es dinámica y las muestras que se pueden obtener para el consumo recreacional podrían provenir de variedades de cannabis cultivadas en diferentes pisos térmicos y regiones de la topografía colombiana, o provenientes de cultivos hidropónicos o del uso de semillas pertenecientes a distintas variedades o cepas, entre otros aspectos. En segundo lugar, en el presente estudio no se encontraron cannabinoides sintéticos (moléculas diseñadas en un laboratorio que producen efectos similares al THC por unión a los receptores CB1 y CB2 del sistema endocanabinoide), del tipo *new psychoactive substances* (NPS). Este resultado es relevante ya que este tipo de moléculas sintéticas no están fiscalizadas en las convenciones de drogas de abuso de 1961 ni de 1971^39^ y, por consiguiente, no tienen estudios de seguridad y eficacia, lo cual constituye un riesgo relevante en salud pública al ser incluso más tóxicas que el cannabis utilizado con fines recreacionales. En tercer lugar, en todas las muestras analizadas se encontró THC en diferentes concentraciones, las cuales indiscutiblemente están asociadas a diferentes riesgos toxicológicos que se pueden ver exacerbados dependiendo de la frecuencia de consumo que tengan cada uno de los usuarios^32^. Gracias a los efectos combinados del THC como psicoactivo y depresor del sistema nervioso central, esta molécula causa relajación, alteraciones de la percepción y de la sensación de bienestar, efectos que buscan los consumidores sociales de este tipo de droga^40^. Finalmente, como cuarto aspecto a destacar es que, en 8 de las 87 muestras analizadas, se encontró una concentración similar o mayor de CBD comparada con THC, razón por la cual fueron clasificadas como quimiotipo II. Este hallazgo es notable teniendo en cuenta que el CBD es no psicoactivo y mitiga los efectos adversos del THC^41^. Este tipo de muestras puede provenir de cultivos destinados al uso medicinal del cannabis con posterior tratamiento de los extractos para disminuir al máximo la concentración de THC, de la creación de cepas con contenidos de CBD modificados o de otras dinámicas de cultivo y producción que buscan disponer una oferta de cannabis con mayor concentración de CBD, tanto para uso recreativo como terapéutico.

A la fecha, en Colombia existe un estudio similar a la presente investigación. En 2009, Florian *et al*. cuantificaron cannabinoides en muestras de material fresco de marihuana cultivadas en cuatro regiones de Colombia: Llanos orientales, Santa Marta, Cauca y Eje Cafetero^42^. En las cuatro regiones se encontró como cannabinoide principal el THC con mayores concentraciones en las muestras provenientes de Llanos Orientales (hasta un 17,6%) y Cauca (hasta un 15,5%), lo que permitiría sugerir que, con el transcurso de los años, y comparando con el presente estudio, en Colombia se están cultivando variedades de Cannabis fitomejoradas, ya que los contenidos máximos de THC encontrados en las muestras de material vegetal de la ciudad de Medellín, alcanzan concentraciones hasta del 21,3% y, a nivel mundial, el contenido de THC sin manipulación genética, no supera el 7%^43^. Este hallazgo se corrobora en el estudio del Ministerio de Justicia y del Derecho, la Dirección de Política de Drogas y el Observatorio de Drogas de Colombia en el 2013 cuando afirman que: “el tetrahidrocannabinol (THC), es el cannabinoide más abundante entre los cannabinoides vegetales en Colombia”^38^. En cuanto al CBD en el estudio de Florian se encontraron concentraciones máximas de 4,9% que en ninguna de las muestras cuantificadas superaron las concentraciones de THC. En este estudio la concentración (% p/p) de CBD máxima encontrada fue de 9,5% y en algunos casos los valores fueron superiores a las concentraciones para THC. Lo anterior presume un uso recreacional de variedades fitomejoradas ricas en CBD lo cual podría ser un indicio de cambio en las dinámicas de consumo dentro de la ciudad, que favorecen los cuadros tóxicologicos cuando solo se consume cannabis con altos contenidos de THC sin CBD. El CBD al no poseer actividad psicotrópica tiene efectos neuroprotectores, antiinflamatorios y ansiolíticos, de igual manera existen estudios que indican que podría atenuar algunos de los efectos neurocognitivos y conductuales del THC^44^^,^^45^^,^^46^; y aunque la evidencia de interacción entre ambos componentes no es concluyente por la existencia de estudios contradictorios^47^^,^^48^, la presencia de CBD en cannabis de uso recreativo se perfila como un posible factor a controlar, con el propósito de reducir los riesgos toxicológicos asociados al consumo^41^^,^^49^. Siguiendo esta línea, se observa que, en ensayos clínicos realizados para personas consumidoras de cannabis, las farmacoterapias basadas en CBD (cannabis con 0,4% de THC y 9% de CBD) han reducido la frecuencia de consumo de cannabis, las ansias y los síntomas de abstinencia^50^. De esta manera, el material rico en CBD es pensado como una alternativa de mitigación de riesgos y resultados adversos en el consumo de cannabis, e incluso en usuarios de otras drogas ilícitas y alcohol, y en la reducción de adicción a opiáceos^51^.

En el campo internacional, un estudio^52^ llevado a cabo en 2021, en Innsbruck, Austria, sobre 93 muestras de marihuana incautadas, reveló que todas las muestras tenían THC; sin embargo, un 45% de ellas presentaron concentraciones mayores de CBD (entre el 2,5% y el 14%) con respecto al THC. Lo anterior, pone en evidencia el uso de muestras de marihuana con fines recreativos ricos en CBD y que, según los resultados de este estudio, es una práctica que empieza a ser empleada en la ciudad de Medellín. Adicionalmente, en el estudio austríaco se detectó un cannabinoide sintético y 15 pesticidas. En el presente estudio, como se mencionó previamente, no se detectaron cannabinoides sintéticos ni pesticidas volátiles, por lo menos en altas concentraciones.

Teniendo en cuenta las concentraciones de THC encontradas en las diferentes muestras analizadas y de acuerdo a la evidencia académica existente, las variedades de marihuana circulante en la ciudad y que presentaron altos contenidos de THC (mayor a dos dígitos) son predominantemente las clasificables como c*reepy*, para distinguirlas de las variedades convencionales; “la *creepy* posee un porcentaje de THC mayor (entre el 10% y el 25%) al de la variedad regular, además de que permite dos o más cosechas al año, lo que incrementa su rentabilidad, donde la mayor cantidad de THC se debe a modificaciones genéticas”^53^. Este fenómeno se ajustaría a las tendencias continentales por incrementar el nivel de THC en la marihuana en países productores como Paraguay, Uruguay, México, Costa Rica y Colombia^53^. 

La marihuana con una alta composición porcentual de THC genera dosis de alta potencia que hacen efecto con una o dos “inhalaciones” al fumar, aumentando la rapidez con que aparecen los efectos^54^, el problema radica en que una marihuana más potente supone un mayor riesgo de intoxicación, y un mercado desregulado limita la posibilidad que tienen los consumidores en la práctica de calibrar o decidir su dosis de THC. 

Fumar marihuana con los niveles de THC descritos no es una práctica inocua, sumado a que los fumadores inhalan profundamente y mantienen la respiración para maximizar la absorción del THC, incrementando los riesgos de intoxicaciones leves, graves o agudas^55^. 

El THC, como constituyente principal de la marihuana circulante en Medellín, se ajusta a las tendencias y preferencias de productores y consumidores a nivel continental. La literatura académica y los resultados de la investigación evidenciarían una confluencia entre las variedades genéticamente modificadas (*creepy*) para obtener mayores niveles de THC y el incremento en la propensión a un mayor consumo; dicho de otro modo, esta confluencia de oferta y demanda mostraría una inclinación por efectos depresores más fuertes sobre el sistema nervioso de los consumidores por medio de dosis más potentes^53^^,^^56^.

Los riesgos de esta concurrencia fáctica es que las probabilidades de intoxicaciones se incrementan, arrastrando consigo mayores niveles de morbilidad para consumidores que no pueden decidir en un mercado ilegal que impide controlar los niveles de THC deseados. Una regulación técnica y científicamente estructurada del comercio de cannabis en la región podría permitir el control de los quimiotipos disponibles, permitiendo dinámicas de consumo que disminuyan el riesgo toxicológico de la sustancia y su respectivo impacto en la salud pública.

Es necesario ahondar con la caracterización y análisis químico de la marihuana circulante de una manera continua y permanente, al igual que con las demás sustancias de uso ilícito, con la finalidad de aportar evidencia científica que posibilite resignificar los marcos normativos, valorativos y teóricos sobre los que se fundamenta el abordaje convencional a la problemática del consumo de psicoactivos y las políticas antidrogas, en ciudades y países de la región.
