# Supplementary material for: Cannabis recreativo: Perfil de los cannabinoides presentes en muestras de marihuana suministradas por población consumidora
Source: Salud Colect. 2023 Mar 30;19:e4385. [Article in Spanish] doi: 10.18294/sc.2023.4385 (PMC11961222; doi:10.18294/sc.2023.4385)
Supplement: Translation

# Translation: Recreational cannabis: Profile of cannabinoids present in marijuana samples supplied by the consuming population

**Authors:** Santiago Gómez Velásquez, Ángela María Amaya Heredia, Santiago Bedoya Moncada, Juan Esteban Patiño González, Jorge Ariel Martínez Ramírez

**Affiliations:** 1 Master’s Degree in Public Health. Secretariat of Youth of Medellín. PhD Candidate in Epidemiology and Biostatistics, Universidad CES, Medellín, Colombia.sagomez@ces.edu.co; 2 Pharmacuetical Chemist. Universidad Nacional de Colombia, Bogotá, Colombia.aamayah@unal.edu.co; 3 Undergraduate Degree in Political Science. Secretary of Youth of Medellín, Medellín, Colombia.santiago.bedoyamon@gmail.com; 4 Master’s Degree in Psychology. Secretariat of Youth of Medellín, Medellín, Colombia. juan.patinog@udea.edu.co; 5 PhD in Forensic Toxicology. Professor, epartamento f Pharmacy, Faculty of Sciences, Universidad Nacional de Colombia, Bogotá, Colombia. jamartinezra@unal.edu.co

**Keywords:** Cannabis, Cannabinoids, Tetrahydrocannabinol, Cannabidiol, Colombia

## Abstract

As cannabis/marijuana is one of the most consumed psychoactive substances in the world, knowing the composition and type of cannabis sold in urban environments is a necessary input for the design of public health policies based on scientific evidence. This study characterized the main phytocannabinoids of marijuana samples (cigarettes or buds) obtained in urban and rural areas of the city of Medellín in October 2021. Non-probabilistic convenience sampling was carried out in which 87 marijuana samples donated by consumers were collected at different collection points throughout the city, and gas chromatography-mass spectrometry and flame ionization techniques were employed for the characterization of phytocannabinoids. Tetrahydrocannabinol (THC) was found to be the main constituent of circulating marijuana in Medellín, where 67.8% of the samples had a high or higher toxicological range for THC; the foregoing in a context where the deregulated market in practice limits the possibility that consumers have to calibrate or decide the concentration of cannabinoids in their doses.

## INTRODUCTION

*Cannabis Sativa L*, also known as cannabis or marijuana, is an ancient plant that is today cultivated all around the world. It has been widely used for both medicinal and recreational purposes. As per the United States Code, the term cannabis or marijuana refers to all parts of the *Cannabis L.* plant, whether growing or not, including the seeds, resin extracted from any part of the plant, and any compound, manufacture, salt, derivative, mixture, or preparation of the plant, its seeds, or resin.^1^ This plant belongs to the Cannabaceae family, and according to the most recent classifications, it is known to have around 170 species.^2^ The Cannabis species is the most well-known, capable of synthesizing approximately 565 substances, 120 of which are molecules with a terpenophenolic skeleton consisting of 21 carbon atoms called phytocannabinoids. The cannabinoid found in the highest proportion in the plant is delta 9-tetrahydrocannabinol (Δ9-THC or THC) and it is responsible for the majority of the psychoactive effects of cannabis. Other phytocannabinoids that also exhibit psychotropic activity but are present in lower proportions in the plant material include cannabivarin (THCV) and CBN, the latter being evaluated in this study as a marker of THC oxidation. There are also other cannabinoids found in the plant, such as cannabidiol (CBD), cannabichromene (CBC), and cannabigerol (CBG), which do not have psychoactive effects and can be found in varying concentrations depending on the strain.^3^^,^^4^

Cannabis is native to tropical environments in central and eastern Asia and has been used by humans since ancient times, adapting to different climatic conditions. Today it is a crop that spans a large part of the world.^5^ When cannabis is consumed, it activates the receptor system and neurotransmitters within the body called the endocannabinoid system (ECS). The THC cannabinoid binds to CB1 and CB2 receptors, generating a wide range of effects, some more desirable than others. For example, THC can help reduce pain and improve appetite; however, it can also cause paranoia and anxiety. On the other hand, CBD reduces the signaling of endocannabinoids, a response that in turn depends on the dose as it binds to the allosteric site of the CB1 receptor, altering the potency of other primary ligands and producing opposite effects.^6^^,^^7^

The most notable psychotropic effects reported by recreational users of THC include pleasure, relaxation, happiness, and increased sensory perception, among others.^8^ However, these effects are accompanied by undesirable effects on the central nervous, respiratory, and cardiovascular systems and certain psychiatric conditions such as depression, sedation, psychotic symptoms, increased cardiovascular activity, exacerbation of preexisting mental illnesses, and symptoms of dependence.^9^

The amount of THC in cannabis samples may decrease through interconversion to cannabinol (CBN), a cannabinoid that is not naturally present in the plant and with very low psychoactive potency, generated through heating or oxidation of the plant material. High concentrations of CBN in cannabis samples may indicate prolonged storage or mishandling of the sample.^10^

Regarding the cannabinoid CBD, plant extracts and derivatives rich in this cannabinoid have been used in therapies for chronic pain, respiratory diseases, certain types of cancer, and cardiometabolic risk, among other conditions.^11^ The variation in effects depends on the quantity and concentration of cannabinoids present in the plant material and its derivatives utilized by users.

Traditionally, and depending on the quantity of these components, *Cannabis sativa* can be classified into three chemotypes. Chemotype I is rich in the psychotropic cannabinoid THC; the majority of modern cultivars belong to this category. These plants are highly appreciated by recreational consumers seeking a “high” and by those using marijuana for holistic purposes. Chemotype II offers a balanced proportion of THC and CBD. Cultivars with this balance are gaining popularity among both recreational and holistic consumers. It produces a strong psychotropic effect, but a similar amount of CBD attenuates the influence of THC and may reduce its harmful psychotropic effects. Chemotype III is rich in CBD and has low THC content. As a result, these varieties produce minimal psychotropic effects; for some recreational and holistic consumers, this lucid effect is highly useful and functional.^4^ Although this classification is not valid from a taxonomic standpoint of the seed, it is practical from an analytical, pharmacological, and legal perspective (in the case of Colombia) as it quickly allows for the identification of the type of sample based on its concentration and its association with toxicological and legal risks. In Colombia, material is considered psychoactive when the THC concentration exceeds 1%.^12^

The psychotropic effects of cannabinoids present in marijuana make it one of the most widely consumed psychoactive substances worldwide, whether its consumption is legal or prohibited. Evidence indicates that the prevalence of marijuana consumption has been increasing over the past decades, with higher rates of use among young people and young adults; indeed, use of the substance continues to occur in different regions, regardless of the regulatory framework with respect to its use.^13^^,^^14^

Something similar can be seen in the American continent. The most recent information reveals that marijuana consumption has increased in eight out of the eleven countries that reported data; according to reports from the Organization of American States (OAS), consumption is occurring at increasingly younger ages, and the overall perception of risk associated with consumption has decreased, with changes in habits and forms of consumption.^15^

In Colombia, the latest National Survey on the Consumption of Psychoactive Substances conducted in 2019 by the National Administrative Department of Statistics [Departamento Nacional de Estadística] (DANE) revealed - in line with global and continental trends - that the highest lifetime prevalence of consumption of illicit substances was found for marijuana, at 8%. It is important to note that Colombian youth, consistent with age trends in the continent, also report an increasingly early onset of marijuana use and a visible inclination to experiment with a perceived mild level of risk.^16^ Similarly, the evidence unambiguously indicates an increase in the consumption of substances classified as illegal (marijuana) and a decrease in the consumption of legal substances (tobacco) among the Colombian population.^15^^,^^17^ The supply reduction approach that has for years been implemented by the Colombian State has not reduced consumption; research on this matter establishes consistently, over three decades of study, that Colombians perceive there to be almost no barriers to accessing substances such as marijuana.^18^

In addition, scientific literature highlights that, since 1992 and up to the latest national survey in 2019, the city of Medellín presents a higher prevalence of psychoactive substance than the national average.^18^ This result is corroborated by local studies such as the “El Análisis de la Situación de Salud 2005-2015” [Health Situation Analysis 2005-2015] published by the city government, which states: “The municipality of Medellín has a higher lifetime prevalence than the country as a whole in the consumption of alcohol, tobacco, marijuana, cocaine, *basuco* [cocaine base paste], tranquilizers, stimulants, heroin, ecstasy, and in general, any legal, illegal, or illicit substance.” Additionally, young people in the city have a higher lifetime prevalence of marijuana use compared to population groups in other age ranges.^19^^,^^20^

As can be inferred from the above, the actions of national and municipal institutions to affect the demand and use of psychoactive substances, in general, and marijuana, in particular, have not generated significant changes in consumption patterns. Additionally, the different studies on cannabis abuse mainly focus on the frequency of use,^21^ neglecting the composition and concentration of cannabinoids that different samples may have. This situation is concerning considering that the toxicological risk definitely changes according to the concentration of the plant’s main psychoactive component. Debates surrounding the legalization of recreational marijuana use and its medicinal use continue to shape the political agendas in Colombia and different countries in the region, which could indicate a transition from a prohibitionist paradigm towards strategies based on preventive medicine, public health, and harm reduction.^22^

Identifying the types of cannabinoids present in marijuana samples circulating in urban environments is essential for developing comprehensive care strategies in public health and specialized medicine that can guide preventive interventions regarding the issues associated with the consumption of psychoactive substances. Such an analysis simultaneously provides the opportunity to strengthen or redefine the approach of promotion and prevention programs in addressing risk behaviors, basing their design and implementation on scientific evidence.

In Colombia, through Act 1787 of 2016, a regulatory framework was created to allow safe and informed access for the medical and scientific use of cannabis and its derivatives. In this regard, according to Decree 613 of 2017 issued by the Ministry of Health and Social Protection, cannabis material is considered psychoactive if the THC concentration is greater than 1% in dry weight. This indicates that for cannabis derivatives, at minimum it is necessary to quantify the cannabinoids THC as the psychoactive component, CBD as the medicinal component, and CBN as an indicator of THC degradation. Based on this regulation, the main objective of this study was to determine the concentration of THC, CBD, and CBN in cannabis samples (plant material) collected voluntarily in six *comunas* [urban subdivisions] and two *corregimientos* [rural subdivisions] of the district of Medellin. This study also aimed to provide a general sociodemographic characterization of recreational cannabis consumption.

## MATERIALS AND METHODS

A cross-sectional study with prospective data collection and an analytical scope was carried out; non-probabilistic sampling was used to capture cannabis users in the city of Medellin who voluntarily agreed to donate they substance they consume as a sample for analysis. This research presented no risks to the participants, as established by Resolution 8430 of 1993, and complied with the relevant bioethical code in the data collection process. Each participant was asked to complete a survey-like form in order to collect sociodemographic information and consumption frequencies, which did not identify participants nor inquire about sensitive aspects of their behavior. Participants answered without providing their signature but did give verbal consent, an approach that took into account the fear among substance users of being identified, stigmatized, or possibly facing legal consequences. The collection and subsequent handling of the samples was approved by the International Center for Strategic Studies against Narcotrafficking [Centro Internacional de Estudios Estratégicos contra el Narcotráfico] (CIENA) of the National Police and by the National Narcotics Fund [Fondo Nacional de Estupefacientes]. To fully preserve the anonymity of the study participants, neither entity had direct or indirect contact with participants, as any practices that could be perceived as identification or tracing were avoided.

### Sample collection

The collection of samples was carried out by the company Consultoría Especializada en Drogas, Salud & Sociedad (CEDSS), contracted by the Universidad Nacional de Colombia, Bogotá Campus. All samples were collected in the district of Medellin during the month of October 2021, through 22 interventions at different social events targeting youth, in which a booth was set up to encourage cannabis users to approach of their own accord and voluntarily donate the substance they consume. Detailed protocols were followed at the time of each collection to ensure standardization and quality in the collection process. Each user was asked to optionally fill out an form that allowed for the collection of information regarding the sample and general sociodemographic data. Each participant was provided with information about the research, its objectives, and the use of the resulting information for strictly academic and research purposes, then asked for their verbal consent to complete the form and voluntarily donate the sample. During the different collection sessions, four types of sampling were conducted: snowball sampling, convenience sampling, quota sampling, and voluntary or self-selected participant sampling. Once all the samples were collected, they were transported by officials from the CIENA to the Pharmaceutical Instrumental Analysis Laboratory of the Department of Pharmacy at the Universidad Nacional de Colombia, where the respective chromatographic analyses were carried out.

### Types of samples and preparation for analysis

As a selection criterion for sample collection, it was determined that only plant material would be included. Other forms of cannabis were excluded from this study. The samples were collected in the *comunas* of Laureles-Estadio, La Candelaria, Poblado, San Javier, Guayabal, Robledo, and the *corregimientos* of San Antonio de Prado and Santa Elena in the district of Medellin during the month of October 2021.

Each of the cannabis plant material samples was macerated until a homogeneous particle size was achieved. A portion (50 mg) of the macerate was used for cannabinoid extraction by suspending it in 1.5 mL of ethanol in an Eppendorf vial, followed by three minutes of vortexing, five minutes of sonication, and five minutes of centrifugation at 9,000 rpm. The supernatant was filtered into a chromatography vial using a 0.45 µm membrane, and 1 µL was injected into each of the gas chromatographs.

### Chemical anaylsis technique

For the identification of the different cannabinoids, gas chromatography with mass spectrometry and electron impact (GC-MS/EI) was used for all samples, while gas chromatography with flame ionization detection (FID) was employed for quantification. Both analytical methodologies were validated following the validation guidelines of the United Nations Office on Drugs and Crime (UNODC).^23^

The parameters for the identification of different cannabinoids using the GC-MS/EI analytical methodology were taken from the UNODC document “Recommended Methods for the Identification and Analysis of Cannabis and Cannabis Products.”^24^

GC-MS/EI. The system used for the identification of different phytocannabinoids employed a Thermo TRACE 1300 gas chromatograph with an ISQ QD detector and a TR-5MS analytical capillary column (30 m x 0.25 mm i.d x 0.25 µm). The injector, detector, and transfer line temperatures were set at 290°C, 230°C, and 280°C, respectively. The injection was performed in a split 20:1 mode, and the oven was programmed to start at 240°C for one minute, with a ramp of 12°C/min up to 270°C for five minutes. The Chromeleon® software with the NIST 2007 Mass Spectral Library was used for data evaluation.

GC-FID. The quantification of phytocannabinoids in the plant material was performed using a Shimadzu GC-2010 Plus gas chromatograph equipped with a Shimadzu AOC-20i autosampler and an analytical capillary column (30 m x 0.25 mm x 0.25 µm, Restek, Bellefonte, Pennsylvania, US). The detector and injector temperatures were maintained at 290 °C and 300 °C, respectively. A 1 µL aliquot of the cannabis extract dissolved in 99% ethanol was injected in a split 10:1 mode. The oven was programmed to start at 200 °C for two minutes with a ramp of 10 °C/min up to 260 °C for seven minutes, resulting in a total run time of 15 minutes. Quantification was performed for THC, CBD, and CBN using tetracosane as an internal standard at a concentration of 100 ppm. The LabSolutions VR 5.52 software from Shimadzu was used for data evaluation.

### Chemical and reactive substances

 The reference material was “THC cannabinoids mixture-3” with delta-9-tetrahydrocannabinol (Δ9-THC), cannabinol (CBN), and cannabidiol (CBD) at a concentration of 1 mg/mL from Cerilliant Corporation (Round Rock, TX) and tetracosane of 99% purity from Sigma/Merck (Colombia) as well as analytical-grade ethanol at 99% purity from PanReac. The working solution of cannabinoids was prepared at a concentration of 100 µg/mL.

### Statistical analysis

The analysis of the information was conducted using the open-source statistical software Jamovi 2.3.21. Categorical variables were analyzed by calculating absolute and relative frequencies. Descriptive statistics including measures of central tendency (mean and median) and measures of dispersion (standard deviation and interquartile range) were used for quantitative variables.

## RESULTS

A total of 87 cannabis samples were collected, each corresponding to plant material, along with an equal number of survey responses. The average amount donated for this material was 282.8 mg, with a range of 29.1 to 1,037.4 mg. 

Regarding the sociodemographic characteristics of the sample donors, as shown in Table 1, it can be observed that 22 were female and 63 were male, while 2 individuals did not respond to the sex category. The majority of the sample donors had a professional or postgraduate education level and fell within the age range of 29 to 59 years. It is also worth highlighting that 70% of the sample donors belonged to the middle or high socioeconomic level (stratum 3-4 or stratum 5-6), according to the system of socioeconomic stratification defined in Colombia.^25^ Furthermore, 80.5% of the consumers had been using the substance for more than 5 years.

**Table 1:** Characteristics of the participants who donated samples (n=87). Comunas and corregimientos of Medellín, Colombia, 2021.

Characteristics	Total		Women		Men	
	n	%	n	%	n	%
Education						
None	3	3.5	0	0.0	3	4.8
Primary school	1	1.2	0	0.0	1	1.6
Secondary school	15	17.2	1	4.6	14	22.2
Technical Degree	17	19.5	4	18.2	13	20.6
Professional	40	46.0	17	77.3	23	36.5
Graduate	8	9.2	0	0.0	8	12.7
No response	3	3.5	0	0.0	1	1.6
Age						
18-28	29	33.3	7	31.8	22	34.9
29-59	55	63.2	15	68.2	40	63.5
> 60	1	1.2	0	0.0	1	1.6
No response	2	2.3	0	0.0	0	0.0
Socioeconomic level*						
Low	24	27.6	2	9.1	22	34.9
Medium	53	60.9	19	86.3	34	54.0
High	8	9.2	1	4.5	7	11.1
No response	2	2.3	0	0.0	0	0.0
Period of use						
1 to 2 years	2	2.3	0	0.0	2	3.2
3 to 5 years	11	12.6	2	9.1	9	14.3
Over 5 years	70	80.5	19	86.4	49	77.8
No response	4	4.6	1	4.6	3	4.8

Source: Own elaboration. Note: Two of the participants who donated samples did not respond to the sex category. *Based on the socioeconomic stratification of residential dwellings of municipalities carried out according to the Regimen of Domiciliary Public Services in Colombia in Act 142 of 1994.

### Chromatographic analysis

The 87 samples collected within the framework of the Youth Public Health Program of the Secretary of Youth in Medellin were obtained in six of the sixteen *comunas* of the urban area of the city, as well as two of the five *corregimientos* that make up the rural zone. According to Table 2, the urban territories contributed 83.9% (73) of the samples, while the rural zone contributed 16.1% (14).

**Table 2:** Places from which samples of cannabis plant material were collected (n=87). Comunas and corregimientos of Medellín, Colombia, 2021.

Comuna or corregimiento	n	%
Laureles-estadio	27	31.0
La candelaria	18	20.7
Poblado	12	13.8
Santa Elena	8	9.8
San Javier	7	8.1
San Antonio de prado	6	6.9
Guayabal	5	5.8
Robledo	4	4.6

Source: Own elaboration.

Of the six *comunas* from which samples were collected, the *comunas* located on the western side (Laureles, Guayabal, Robledo, and San Javier) contributed 58.9% of the samples. The *comunas* located in the eastern part of the city (La Candelaria and Poblado) contributed 41.1%. The *corregimiento* of San Antonio de Prado provided 42.8% of the samples from the rural zone. The number of gathered samples and their spatial dispersion allowed for a comprehensive overview of the cannabinoids present in the marijuana consumed in the city of Medellín.

### Analysis of the samples

#### GC-MS/EI

The qualitative analysis by GC-MS/EI allowed for the detection of seven phytocannabinoids, ordered according to their incidence in the different samples (Table 3): tetrahydrocannabinol (THC), cannabigerol (CBG), cannabinol (CBN), cannabichromene (CBC), delta-9-tetrahydrocannabivarin, cannabidiol (CBD) and cannabidivarin (CBDV).

**Table 3:** Cannabinoids present in the samples of cannabis plant material (n=87). Comunas and corregimientos of Medellín, Colombia, 2021.

Cannabinoides	n	%
THC (tetrahydrocannabinol)	87	100.0
Cannabigerol (CBG)	78	89.5
Cannabinol (CBN)	67	77.0
Cannabicromene (CBC)	63	72.4
Delta-9-Tetrahydrocannabivarin	47	54.0
Cannabidiol (CBD)	13	14.9
Cannabidivarin (CBDV)	2	2.2

Source: Own elaboration.

The structure and composition of each of the phytocannabinoids can be observed in Table 4. The presence of THC in 100% of the samples and CBG in 78 of them is logical, considering that THC is the largest component of cannabinoids in plant material, and CBG is derived from CBG acid, the first phytocannabinoid biosynthesized after the formation of olivetolic acid, serving as a precursor to other cannabinoids in the plant.^26^ However, despite THC having an average composition of 89.4% of the total cannabinoids in the 87 samples, the therapeutic non-psychoactive cannabinoid CBD had an average composition of 37.1%, which is a significant percentage in the total composition of the 13 samples in which it was present. The average presence of other cannabinoids (CBG, CBN, CBC, CBDV, and delta-9-tetrahydrocannabivarin) was less than 2.3%.

**Table 4:** Percentage presence of each cannabinoid found with respect to all the cannabinoids quantified in the samples of cannabis plant material (n=87). Comunas and corregimientos of Medellín, Colombia, 2021.

Cannabinoid	Molecular structure	Positive samples	Composition		
			Mean ± SD	Median	(Q1 - Q3)
THC (Tetrahydrocannabinol)		87	89.4 ± 15.8	94.9	92.1 - 95.9
CBG Cannabigerol		78	2.3 ± 1.7	1.9	1.5 - 2.5
CBN Cannabinol		67	1.8 ± 2.0	1.1	0.8 - 1.9
CBC Cannabicromene		63	1.8 ± 1.1	1.4	1.2 - 2.0
Delta-9-Tetrahidrocannabivarin		47	0.5 ± 0.4	0.4	0.3 - 0.6
CBD Cannabidiol		13	37.1 ± 26.1	51.3	6.9 - 57.0
CBDV Cannabidivarin		2	0.9 ± 0.9	0.9	0.6 - 1.3

Source: Own elaboration.

Regarding their psychotropic activity,^27^ this study found THC and CBN (low activity) as psychoactive cannabinoids, while CBG, CBC, CBD, and CBDV were identified as non-psychoactive components. In comparison to other cannabinoids, THC made up a significantly higher percentage of the composition. Therefore, when studying the composition of cannabis in the city of Medellín, THC should be considered as the main contributor to the type of effect that consumers expect from the substance. CBN, another less psychoactive component of cannabis - ten times less potent than THC^28^ - was present in 77.0% of the samples (Table 3). However, its concentration in the samples did not exceed 1%, indicating that all 87 analyzed samples corresponded to fresh plant material with minimal manipulation.

Regarding non-psychoactive cannabinoids, CBD was identified in 13 samples with a significant percentage compared to other cannabinoids (37.1%). CBD structurally differs from THC by the presence of a carbon double bond and a hydroxyl group, which prevents the deleterious effects of high doses of THC, moderating its psychoactive effects while exhibiting medicinal properties.^29^ Additionally, CBG was identified in 78 samples with an average presence of 2.3% (Table 4). These two cannabinoids, acting through a mechanism different from THC, potentially have certain anti-inflammatory, analgesic, antipsychotic, anti-ischemic, antiepileptic, and anxiolytic effects that are being widely studied.^30^^,^^31^

Table 5 reports the chemotype and average concentration of THC, while Table 6 shows the percentage of each of the three most relevant cannabinoids in this study.

**Table 5:** Chemotype and toxicological risk given the THC present in the analyzed cannabis samples (n=87). Comunas and corregimientos of Medellín, Colombia, 2021.

Comuna or corregimiento	Chemotype				Toxicological Risk (THC)*									
	I		II		Extremely high		Very high		High		Medium		Low	
	n	%	n	%	n	%	n	%	n	%	n	%	n	%
San Javier	7	100.0	0	0.0	0	0.0	0	0.0	2	28.6	3	42.9	2	28.6
Robledo	4	100.0	0	0.0	1	25.0	0	0.0	2	50.0	1	25.0	0	0.0
La Candelaria	17	94.4	1	5.6	0	0.0	3	16.7	9	50.0	5	27.8	1	5.6
Laureles-Estadio	25	92.6	2	7.4	0	0.0	8	29.6	13	48.2	4	14.8	2	7.4
Poblado	12	100.0	0	0.0	1	8.3	3	25.0	5	41.7	1	8.3	2	16.7
Guayabal	5	100.0	0	0.0	0	0.0	3	60.0	1	20.0	1	20.0	0	0.0
San Antonio de Prado	6	100.0	0	0.0	0	0.0	1	16.7	5	83.3	0	0.0	0	0.0
Santa Elena	3	37.5	5	62.5	0	0.0	0	0.0	2	25.0	2	25.0	4	50.0
Total	79	90.8	8	9.2	2	2.3	18	20.7	39	44.8	17	19.5	11	12.6

Source: Own elaboration.

*Range of THC Concentration - Toxicological Risk^32^: low: THC content between 1.0%-5.0%; moderate: THC content between 5.0% - 10.0%; high: THC content between 10.0% - 15.0%; very high: THC content between 15.0% - 20.0%; extremely high: THC content between 20.0% - 25.0%.

**Table 6:** Quantification of THC, CBD and CBN present in the analyzed cannabis samples (n=87). Comunas and corregimientos of Medellín, Colombia, 2021.

Comuna or corregimiento	Sample weight (mg)	THC (% w/w)	CBD (% w/w)	CBN (% w/w)	CBD+THC+CBN (% w/w)
	Mean ± SD	Mean ± SD	Mean ± SD	Mean ± SD	Mean ± SD
San Javier	101.2 ± 37.6	7.6 ± 3.1	0.2 ± 0.1	0.3 ± 0.2	8.1 ± 3.2
Robledo	231.3 ± 115.7	14.7 ± 6.6	0.4 ± 0.3	0.1 ± 0.1	15.2 ± 6.6
La Candelaria	380.6 ± 320.4	11.0 ± 3.7	0.6 ± 1.4	0.1 ± 0.2	11.7 ± 3.2
Laureles-Estadio	278.7 ± 177.1	12.4 ± 4.2	0.8 ± 2.0	0.1 ± 0.1	13.2 ± 3.8
Poblado	260.3 ± 147.2	12.9 ± 5.3	0.2 ± 0.1	0.3 ± 0.6	13.4 ± 5.6
Guayabal	158.7 ± 78.6	13.1 ± 4.2	0.2 ± 0.0	0.3 ± 0.1	13.5 ± 4.3
San Antonio de Prado	198.9 ± 131.0	13.8 ± 2.8	0.2 ± 0.0	0.2 ± 0.1	14.1 ± 2.9
Santa Elena	435.6 ± 295.0	6.1 ± 4.1	4.4 ± 3.5	0.0 ± 0.0	10.5 ± 2.3
Total	282.8 ± 223.4	11.5 ± 4.7	0.8 ± 2.0	0.1 ± 0.3	12.4 ± 4.2

Source: Own elaboration.

According to current Colombian legislation, all quantified samples would be classified as psychoactive material because the concentration of THC exceeds 1% w/w (Table 5). The vast majority of the quantified samples (90.8%) correspond to chemotype I, rich in the cannabinoid THC, indicating that they primarily have psychotropic effects. Another 9.2%, corresponding to 8 samples, exhibited chemotype II, rich in CBD (higher CBD content than THC), suggesting that these samples may have fewer psychotropic effects on the body due to the counteractive effects of high concentrations of the medicinal cannabinoid. Specifically, the 8 CBD-rich samples reported CBD contents in the range of 4.7% to 9.6% w/w. The neighborhoods where the highest number of cannabis plant material samples were collected were Laureles-Estadio, La Candelaria, and Poblado. Overall, the majority of the samples (44.8%) present high toxicological risks in relation to the concentration of THC present. 

As shown in Table 6, the average concentrations (% w/w) of THC were found to be in the range of 6.1-14.7% with a mean of 11.5%. The concentrations of CBD ranged from 0.2-4.4% with a mean of 0.8%, and the concentrations of CBN ranged from 0.0-0.3% with a mean of 0.1%. The average concentration of the total sum of these three mentioned cannabinoids was 12.4%.

## DISCUSSION

Despite scientific evidence showing that acute cannabis consumption increases inflammation in the respiratory pathways and damages lung tissue, as well as studies that show that chronic cannabis use is associated with a higher risk of chronic diseases such as bronchitis, emphysema, chronic respiratory inflammation, and impaired respiratory function,^33^ marijuana smoking continues to be a growing practice in Medellín and different cities in the region.^18^^,^^34^^,^^35^^,^^36^^,^^37^ All the adverse effects observed in the respiratory pathways and lung tissue are closely related to the primary method of marijuana consumption. When marijuana is consumed via inhalation, THC reaches peak blood concentrations between seven and ten minutes after consumption, with maximum effects occurring within 20 to 30 minutes, and lasting up to three to four hours, subject to interindividual variability.^38^

Several relevant findings can be highlighted in this characterization of the components of the obtained marijuana samples. Firstly, the cannabinoid content is not consistent across all samples, indicating that the supply of cannabis in the Medellín District is dynamic. The recreational cannabis samples could originate from different varieties of cannabis cultivated in different thermal floors and regions of the Colombian topography, or from hydroponic crops or the use of seeds belonging to different varieties or strains, among other factors. Secondly, synthetic cannabinoids (molecules designed in a laboratory that produce THC-like effects by binding to the CB1 and CB2 receptors of the endocannabinoid system), such as new psychoactive substances (NPS), were not found in this study. This result is relevant because these types of synthetic molecules are not regulated by the 1961 or 1971 drug control conventions^39^ and therefore lack safety and efficacy studies, posing a significant public health risk as they can be even more toxic than cannabis used for recreational purposes. Thirdly, THC was found in different concentrations in all analyzed samples, which is unquestionably associated with varied toxicological risks that can be exacerbated depending on the frequency of use by each user.^32^ Thanks to the combined effects of THC as a psychoactive and central nervous system depressant, this molecule causes relaxation, alterations in perception, and a sense of well-being, which is sought after by social consumers of this type of drug.^40^ Lastly, as a fourth aspect worth highlighting, in 8 out of the 87 analyzed samples, CBD was found in concentrations similar to or higher than that of THC, and were therefore classified as chemotype II. This finding is noteworthy considering that CBD is non-psychoactive and mitigates the adverse effects of THC.^41^ These types of samples may come from crops intended for the medicinal use of cannabis with subsequent treatment of extracts to minimize THC concentration, from the creation of strains with modified CBD content, or from other cultivation and production dynamics aiming to provide cannabis with higher CBD concentrations for both recreational and therapeutic use.

To date, there is one study in Colombia similar to the present investigation. In 2009, Florian *et al.* quantified cannabinoids in samples of fresh marijuana material cultivated in four regions of Colombia: Llanos Orientales, Santa Marta, Cauca, and Eje Cafetero.^42^ In all four regions, the main cannabinoid found was THC, with higher concentrations in samples from Llanos Orientales (up to 17.6%) and Cauca (up to 15.5%). This suggests that over the years, and in comparison with the present study, enhanced varieties of Cannabis are being cultivated in Colombia, as the maximum THC content found in the plant material samples from the city of Medellín reach concentrations of up to 21.3%, whereas worldwide, the THC content without genetic manipulation does not exceed 7%.^43^ This finding is corroborated by a study conducted by the Ministry of Justice and Law, the Drug Policy Directorate, and the Drug Observatory of Colombia in 2013, which states that “tetrahydrocannabinol (THC) is the most abundant cannabinoid among plant cannabinoids in Colombia.”^38^ Regarding CBD, Florian’s study found maximum concentrations of 4.9%, which did not surpass the concentrations of THC in any of the quantified samples. In this study, the maximum CBD concentration (% w/w) found was 9.5%, and in some cases, the values were higher than the concentrations for THC. This suggests recreational use of enhanced varieties rich in CBD, which could be an indication of a change in consumption dynamics within the city, creating more favorable toxicological conditions in comparison to when cannabis with high THC content is consumed without CBD. CBD, due to its lack of psychotropic activity, has neuroprotective, anti-inflammatory, and anxiolytic effects. Additionally, studies indicate that it could attenuate some of the neurocognitive and behavioral effects of THC.^44^^,^^45^^,^^46^ Although the evidence of interaction between both components is inconclusive due to the existence of contradictory studies,^47^^,^^48^ the presence of CBD in recreational cannabis is emerging as a possible factor to control in order to reduce the toxicological risks associated with consumption.^41^^,^^49^ Along these lines, it has been observed that in clinical trials conducted with cannabis users, CBD-based pharmacotherapies (cannabis with 0.4% THC and 9% CBD) have reduced cannabis consumption frequency, cravings, and withdrawal symptoms.^50^ Thus, CBD-rich material is considered an alternative for mitigating risks and adverse outcomes in cannabis consumption, even among users of other illicit drugs and alcohol, and in reducing opioid addiction.^51^

Looking internationally, a study^52^ conducted in Innsbruck, Austria in 2021 using 93 confiscated marijuana samples revealed that all samples contained THC. However, 45% of them exhibited higher concentrations of CBD (ranging from 2.5% to 14%) compared to THC. This highlights the use of CBD-rich marijuana samples for recreational purposes, which, according to the results of the present study, is a practice beginning to be employed in the city of Medellín. Additionally, the Austrian study detected one synthetic cannabinoid and 15 pesticides. In the present study, as mentioned previously, no synthetic cannabinoids or volatile pesticides were detected, at least in high concentrations.

Considering the concentrations of THC found in the different samples analyzed and based on existing academic evidence, the predominant varieties of marijuana circulating in the city with high THC contents (in the double digits) can be classified as “creepy,” a term that has emerged to distinguish them from conventional varieties. “Creepy” marijuana has a higher THC percentage (between 10% and 25%) compared to regular varieties and allows for two or more harvests per year, which increases its profitability. The higher THC content is attributed to genetic modifications.^53^ This phenomenon has to do with continental trends to increase the THC level in marijuana in producing countries such as Paraguay, Uruguay, Mexico, Costa Rica, and Colombia.^53^

Marijuana with a high THC composition produces high potency doses that take effect with one or two “hits” when smoked, increasing the speed at which the effects appear.^54^ The problem lies in the fact that more potent marijuana poses a greater risk of intoxication, and a deregulated market limits consumers’ ability to calibrate or choose their THC dosage in practice.

Smoking marijuana with the described THC levels is not a harmless practice. Additionally, smokers inhale deeply and hold their breath to maximize THC absorption, thereby increasing the risks of mild, severe, or acute intoxication.^55^

THC, as the main component of the marijuana circulating in Medellín, aligns with the preferences of producers and consumers at a continental level. The academic literature and the research findings demonstrate a convergence between genetically modified (“creepy”) strains aimed at achieving higher THC levels and an increased propensity in the population for greater consumption. In other words, this convergence of supply and demand shows a preference for stronger depressant effects on the nervous system of consumers through more potent doses.^53^^,^^56^

The risk of this confluence is that the probability of intoxication increases, leading to higher levels of morbidity for consumers who are unable to make decisions within an illegal market that prevents control over the desired THC levels. A technically and scientifically structured regulation of the cannabis market in the region could allow for control over available chemotypes, enabling consumption dynamics that reduce the toxicological risk of the substance and its impact on public health.

It is necessary to continue the characterization and chemical analysis of circulating marijuana in a continuous and permanent manner, and to do the same with other illicit substances. Such actions would contribute scientific evidence for the redefinition of the normative, evaluative, and theoretical frameworks on which the conventional approach to the issue of psychoactive substance use and antidrug policies is based in cities and countries in the region.
